# Can Mandatory Disclosure Policies Promote Corporate Environmental Responsibility?—Quasi-Natural Experimental Research on China

**DOI:** 10.3390/ijerph18116033

**Published:** 2021-06-03

**Authors:** Yue Liu, Pierre Failler, Liming Chen

**Affiliations:** 1 Hunan Institute of Technology, School of Economics and Management, Hengyang 421000, China; liuyue2013@hnu.edu.cn; 2 Economics and Finance Group, Portsmouth Business School, University of Portsmouth, Portsmouth PO1 3DE, UK; 3 School of Finance and Statistics, Hunan University, Changsha 410079, China

**Keywords:** mandatory disclosure, corporate environmental responsibility, difference-in-differences (DID) model

## Abstract

Corporate environmental responsibility (CER) is an important component of the corporate social responsibility (CSR) report, and an important carrier for enterprises to disclose environmental protection information. Based on the corporate micro data, this paper evaluates the effect of a mandatory CSR disclosure policy on the fulfillment of corporate environmental responsibility by adopting the difference-in-differences model (DID) with the release of a mandatory disclosure policy of China in 2008 as a quasi-natural experiment. The study draws the following conclusions: First, a mandatory CSR disclosure policy can promote the fulfillment of CER. Second, after the implementation of a mandatory CSR disclosure policy, enterprises can improve their CER level through two channels: improving the quality of environmental management disclosure and increasing the number of patents. Third, the heterogeneity of the impacts of mandatory CSR disclosure on CER is reflected in three aspects: different CER levels, different corporate scales and a different property rights structure. In terms of the CER level, there is an inverted U-shaped relationship between the CER level and mandatory CSR disclosure effect. In terms of the corporate scale, mandatory disclosure of CSR plays a greater role in large-scale enterprises. In terms of the structure of property rights, mandatory CSR disclosure has a greater effect on non-state-owned enterprises.

## 1. Introduction

Corporate environmental responsibility (CER) is an important component of a corporate social responsibility (CSR) report and an important carrier for enterprises to disclose environment related behavior information. With the development of global economy, the environment is deteriorating day by day, especially by way of the pollution of the air, water and ocean becoming more and more serious, which brings great threat to the survival and development of human beings [1,2]. Environmental problems have become the bottleneck of economic development. In the 1980s, CSR movements began to rise in developed countries in Europe and the United States, including environmental protection issues. Some non-governmental organizations (NGOs) and public opinions involving Greenpeace, environmental protection, social responsibility, human rights, and other issues also constantly call for the connection between social responsibility and trade. CER requires enterprises to take precautions against environmental problems, take the initiative to assume the responsibility of environmental protection, and promote the development and popularization of environmental technology [3,4,5]. Under increasing external pressure and their own development needs, many European and American multinational companies formulate responsibility codes to make necessary commitments to society, or to meet the needs of different interest groups through environment, occupational health, and social responsibility certification [6,7,8].

Although governments attach great importance to CSR, there is no consistent policy on CSR disclosure, especially in terms of mandatory disclosure and voluntary disclosure. In the second half of 2007, ASEAN countries adopted a semi-voluntary disclosure policy [9], i.e., they allowed enterprises to disclose CSR under some restrictive conditions. Before 2013, Malaysia required the disclosure of corporate environmental information in its annual reports so as to disclose CER and promote “Green Malaysia” [10]. India’s company law of 2013 stipulates that in CSR disclosure, enterprises need to disclose environment and other relevant information in the audited annual report [11]. The EU Directive 2014/95/EU on the disclosure of non-financial information by public interest organizations came into force in 2017 [12,13], explicitly stating that non-financial information disclosure should include CER contents. Italy made it mandatory for companies to publish social and environmental reports for the first time in 2018 [14]. As a developing country, China has implemented a series of environmental policies to encourage enterprises to disclose their CSR, especially emphasizing the popularization of environmental awareness and the implementation of environmental protection measures [15,16,17,18]. As in the primary stage of development, China’s stock exchanges did not make mandatory requirements for CSR disclosure until 2007, i.e., they implemented voluntary disclosure. Accordingly, before 2007, less than 3% of China’s listed companies disclosed CSR reports. In order to reduce the opportunities for enterprises to hide environmental pollution, as well as reduce the cost of government supervision, Shanghai Stock Exchange issued the “Guidelines on Environmental Disclosure of Listed Companies on the Shanghai Stock Exchange” (Guidelines for short) in May 2008, which clearly stipulate the disclosure of CSR reports of related companies listed on the Shanghai Stock Exchange. The Guidelines stipulate that relevant companies must disclose environmental information in the form of a temporary announcement, as well as specify the scope of information that must be disclosed by enterprises identified as seriously polluted by the environmental protection department. At the same time, the procedural requirements of environmental information disclosures are clarified. On December 31, 2008, in the “Notice on Doing a Good Job in 2008 Annual Reports of Listed Companies”, three kinds of companies are required to disclose the CSR report, namely, the sample companies of “Shanghai Stock Exchange Corporate Governance Sector”, the companies issuing overseas listed foreign shares, and financial companies. Additionally, other qualified companies are encouraged to disclose CSR voluntarily [19,20,21]. In both mandatory disclosure and voluntary disclosure of CSR, environmental information is regarded as one of the key aspects.

Global environmental challenges have been transformed into practical environmental responsibility management mechanism [22,23,24]. CER is an important component of CSR, which plays an important role in improving environmentally efficient conservation and protection behaviors. In Hexun’s CSR professional assessment system, which is one of the most widely used systems in China, the weight of environmental responsibility is 20% by default; while for the manufacturing industry, the environmental responsibility weight is as high as 30%. The important role of CER is embodied in the following two aspects: on the one hand, CER can significantly promote innovation performance and improve environmental performance [25,26]. On the other hand, CER can promote the green governance of enterprises, with corporate reputation as a mediator [27,28]. Managers report information on environmental management, policies, and impacts to the public, and publicize the positive aspects of such governance to achieve better results [29]. From the perspective of management mechanism, it is not enough for enterprises to only disclose environmental information, and it is necessary to study the quality of CER [30,31]. Peng et al. [25] put forward an operationalization of the corporate environmental reporting credibility concept, identifying possible determinants, relevant measures, and indicators. Referring to the CER measurement system proposed by Li et al. [32], this paper takes the CSR mandatory disclosure stipulated by Shanghai Stock Exchange of China in 2008 as a quasi-natural experiment, using the DID model to study the impact and effect of the CSR mandatory disclosure policy on CER.

The main work and marginal contribution of this paper is to study the impact of mandatory disclosure policies on CER, as well as the mechanism and heterogeneity of the impact. First, the impact of mandatory disclosure of CSR on CER is evaluated. This empirical study shows that mandatory disclosure of CSR can significantly improve CER as a whole, which has a short-term effect. From the analysis of corporate behavior characteristics, mandatory CSR disclosure improves the relevant management level and technical level of enterprises, then promotes enterprises to fulfill CER. The short-term feature of the effect indicates that the policy is the bellwether of the corporate future strategy. After significantly improving the level of corporate environmental responsibility, the whole market threshold rises, and the improving degree weakens. Second, the impact mechanism of compulsory CSR disclosure on CER is realized by enhancing green management and increasing the moderating effect of patent application. By improving the management level and innovating technology, enterprises can achieve a sustainable development strategy, which not only improves the corporate reputation, but also realizes the long-term economic effect. Third, the heterogeneity of the impact of mandatory CSR disclosure on CER is reflected in the CER quantile level, corporate scale, and the difference of the property rights structure, and this heterogeneity is dependent on the discretionary decision-making behavior of enterprises. Regarding the CER level, this study found an inverted U-shaped relationship between the CER level and the effect of mandatory CSR disclosure. Since the environmental protection foundation of enterprises is different, the promotion ability of mandatory CSR disclosure on CER is also different. The promotion speed is first accelerated and then slowed down. For Chinese enterprises, the goal and purpose of non-state-owned enterprises are not consistent with environmental responsibility to a certain extent. The mandatory disclosure policy forces the shareholders and managers to pay more attention to the performance of environmental responsibility. Therefore, the mandatory CSR disclosure can enhance the CER of non-state-owned enterprises more obviously.

The rest of this paper is arranged as follows: the Section 2 is the research design, describing the basic hypotheses, model setting, data, and core variable measurement. The Section 3 is the empirical test of the effect of compulsory CSR disclosure on CER. The effect is obtained by parameter estimation, and the robustness of the parameter results is tested. The Section 4 analyzes the impact mechanism of the mandatory CSR disclosure on CER. The Section 5 is the heterogeneity analysis of the impact of mandatory CSR disclosure on CER. The Section 6 draws the conclusion.

## 2. Research Design

### 2.1. Research Hypotheses

Compulsory disclosure of CSR reports can promote the full disclosure of corporate information, thus encouraging enterprises to increase their environmental protection practices and improve the quality of their environmental responsibility information disclosure. When China was still in the stage of voluntary disclosure, the government did not stipulate the contents of disclosure, so less than 3% of China’s listed companies disclosed CSR reports, and the quality of CSR reports was uneven, lacking effective information that could reflect corporate behavior. With the increasingly serious environmental problems, both the government and the market require enterprises to strengthen environmental responsibility. However, with the pursuit of profit maximization, enterprises largely ignore the environmental responsibilities, therefore the effect of mandatory CSR disclosure is different from that of voluntary CSR disclosure to the listed companies. Compulsory and voluntary disclosure of CSR have different impacts on corporate behavior and strategy [33]. The will of the government is a bellwether of the market to some extent [34]. Compulsory disclosure policies force more enterprises to fulfill their environmental responsibilities, over time guiding them to adjust their decision-making behaviors, accelerate the promotion of environmental protection work, and improve the quality of environmental responsibility information disclosure. Based on the above analysis, this paper puts forward the following hypothesis:

**Hypothesis** **1** **(H1).** *Mandatory CSR disclosure policy can promote enterprises to fulfill CER.*

In the context of mandatory CSR disclosure, in order to meet the disclosure requirements, enterprises will strengthen the performance of environmental responsibility from two aspects, namely management and technology [35,36]. In management, enterprises will increase the emphasis on their own environmental protection concepts and environmental protection goals, and establish an environmental management system, environmental knowledge education and training, as well as an environmental emergency response mechanism to regulate their own behavior. In the technical level, through the investment of green technology innovation, enterprises can produce more green patents and realize their sustainable development strategy [37]. Thus, they tend to strengthen environmental management and develop more patents to fulfill their environmental responsibilities so as to meet the mandatory CSR disclosure requirements. Based on this, this paper puts forward the following hypothesis:

**Hypothesis** **2** **(H2).** *After the implementation of mandatory CSR disclosure, enterprises fulfill their environmental responsibilities by improving the disclosure quality of environmental management and patents, so as to improve the level of CER*.

In the face of a mandatory CSR disclosure policy, enterprises will make decisions based on profit optimization as well as their own attributes and external characteristics, so as to achieve a balance between economic benefits and environmental benefits. Therefore, there is heterogeneity in the impact of mandatory CSR disclosure on CER. Generally speaking, different levels of CER represent different levels of legal awareness, social evaluation, low-carbon technology and output, and green management. When the CER of an enterprise is at a low level, its environmental problems are usually serious and there is more room for improvement. Therefore, the mandatory CSR disclosure can prompt the enterprise to carry out corresponding innovation and improve its environmental responsibility level at a faster speed. When the enterprise’s environmental protection level is improved to a certain extent, its development encounters bottlenecks, such as limited space for improvement of management and technology, so its CER increase speed slows down. Therefore, the promotion of mandatory CSR disclosure to CER may have an inverted U-shaped feature. At the same time, the corporate behavior will also be affected by the nature of property rights. Different natures of property rights lead to different stakeholders, as well as different interest demands of all parties. For example, shareholders of state-owned enterprises will pay more attention to sustainable strategic development [38], while those of non-state-owned enterprises pay more attention to economic benefit maximization, therefore paying less attention to environmental issues than shareholders of state-owned enterprises. The implementation of the mandatory CSR disclosure policy makes shareholders of non-state-owned enterprises pay more attention to the performance of environmental responsibility. Therefore, the CER improvement level of non-state-owned enterprises may be higher than that of state-owned enterprises. Based on this, this paper puts forward the following hypothesis:

**Hypothesis** **3** **(H3).** *The impact of compulsory CSR disclosure on CER is heterogeneous*.

### 2.2. Model Setting

Compulsory disclosure of CSR will have an impact on the behavior of enterprises, forcing them to pay attention to the disclosure of environmental protection information, therefore under the pressure of market competition and supervision there will be differences in the fulfillment of environmental responsibility between enterprises with mandatory disclosure and those without mandatory disclosure. The mandatory CSR disclosure policy implemented by China’s Shanghai Stock Exchange in 2008 can be regarded as a quasi-natural experiment. There are many methods to evaluate the effect of such policies. The difference-in-differences (DID) model can examine the implementation effect of policies comprehensively. Therefore, this paper chooses the DID model to study the effect of mandatory CSR disclosure on CER.

In recent years, the DID model has been mostly used for quantitative assessment of the implementation effect of public policies or projects in econometrics. In general, the large-scale public policy research is different from ordinary scientific research, and it is difficult to guarantee the complete randomness of sample allocation between the policy implementation group and the control group. The experiment of non-randomly assigned policy implementation group and control group is called natural experiment, which has significant features, i.e., there may be ex-ante differences between different sample groups before the implementation of the policy, which will be ignored through simple before-and-after comparison or lateral comparison, leading to biased estimates of the effect of policy implementation. The DID model is based on the data obtained from natural experiments, and it can effectively control the ex-ante differences between research objects and effectively separate the real results of the policy impact [39].

This paper analyzes the impact of mandatory CSR disclosure on CER, meaning relevant variables need to be set based on the policy implementation time. Therefore, samples selected in this paper are the enterprises that disclosed their CSR reports from 2004 to 2012. The enterprises that are subject to mandatory disclosure belong to the treated group, while the enterprises that are not subject to mandatory disclosure belong to the control group. It is expected that the CER scores of the two groups will change significantly after the implementation of mandatory disclosure of CSR. The basic form of the DID model is as follows:

(1) $$CER_{it}=\propto +\beta Treat_{t\;}\times PL_{i}+\sum_{j=1}^{n}\gamma_{j}X_{jit}+\lambda_{i}+v_{t}+\varepsilon_{ikt}$$

where *CER_it_* is the environmental responsibility rating of company *i* in year *t*; *Treat_t_* is the dummy variable of policy implementation, indicating whether the year *t* is the year of policy implementation or later, if yes it is 1, otherwise it is 0; *PL_i_* is a grouping dummy variable, indicating whether the enterprise *i* is the object of a mandatory disclosure policy, if yes it is 1, otherwise it is 0; *Treat_t_* × *PL_i_* is the interaction term between the policy implementation dummy variable and the grouping dummy variable; *β* is the coefficient that this paper focuses on. If *β* > 0, it indicates that the mandatory disclosure policy has a positive impact on corporate environmental responsibility. X*_jit_* is the *j*-th control variable in the t-th year of company *i*; *λ_i_* is the individual fixed effect; *v_t_* is the time fixed effect. This paper studies H1 through Model (1).

### 2.3. Variable Measurement and Explanation

#### 2.3.1. Explained Variables

The explained variable in this paper is the CER score, which will be used as referring to Li et al. [32] to assess the environmental responsibility of enterprises from five dimensions: legal awareness, evaluation, environment-friendly output, low-carbon technology, and green management. The CER score is calculated as follows: First, as data related to enterprise environmental information are largely unavailable, qualitative indicators are usually obtained by content analysis, and quantitative indicators are obtained by weighted aggregation. Second, the qualitative indicators of 2006–2012 come from Chinese Research Data Services Platform (CNRDS) (www.cnrds.com, accessed on 10 April 2021) and CSMAR, including the business research reports, corporate social responsibility reports of listed companies, and so on. Further, the qualitative indicators of 2004–2005 come from the annual reports of the company (www.cninfo.com.cn, accessed on 10 April 2021) and its CSR reports (if any) by the web crawler technology. Third, the Hexun (www.hexun.com, accessed on 10 April 2021) social responsibility scoring system is used to assign weights to the five dimensions respectively to calculate the final CER scores.

Specifically, the dimension of legal awareness accounts for 10% of the weight, mainly indicating whether the enterprise has the awareness to abide by the laws and regulations related to environmental protection, which is reflected by three indicators. The first indicator is whether the enterprise follows the GRI Sustainability Reporting Guidelines, which provides the direction of the code of conduct that enterprises need to abide by. The second indicator is environmental and sustainable development disclosure. The third indicator is whether environmental penalties are imposed. The environmental reputation of an enterprise in the society is reflected by two indicators: the first one is whether it has received environmental recognition; the second is whether it has an environmental advantage.

The dimension of environment-friendly output accounts for 25% of the weight. It primarily observes whether the corporate production and operation activities are environmentally friendly, which is reflected by three indicators: the first indicator is whether there is a circular economy; the second indicator is the availability of environmentally beneficial products; and the third indicator is whether there are pollution emissions.

Low-carbon technology dimension accounts for 25% of the weight, primarily reflecting whether the enterprise saves energy and whether there are measures to reduce three processing wastes, namely, waste gas, waste water, and waste residue.

Green management dimension accounts for 25% of the weight, reflecting the impact of corporate management on the environment. The detection index is whether there is third-party inspection and whether green office is adopted.

In order to ensure the consistency of the scoring, for the two indicators of whether there is environmental penalty and pollution emission, the enterprise is scored as 0 if there is, and 1 if there is not. For all other indicators, the enterprise is scored as 1 if it is affirmative, and 0 if it is not. The weighted score of all the indicators is the CER score.

Based on the above analysis, the environmental responsibility scoring system established in this paper is shown in Table 1.

#### 2.3.2. Policy Variables

In this paper, the implementation date of the mandatory CSR disclosure policy in 2008 is taken as the policy implementation node, and the enterprises that are forced to disclose are taken as the treated group, while those that voluntarily disclose CSR are taken as the control group. In the treated group, the *Treat × PL* value before 2008 is 0 and after 2008 is 1. In the control group, the *Treat × PL* values before and after 2008 are 0. 

#### 2.3.3. Control Variables

For the control variables, this paper refers to Feng et al. [40], mainly considering the internal characteristics of the enterprise: (1) Corporate scale, which is expressed by the logarithm of total assets. Small enterprises have less incentive to reform and be green, thus have less advantage in technological innovation, while large ones have more advantage in technological innovation. (2) The corporate age, which is represented in this paper by the difference between the study year and the year the enterprise was founded. (3) Capital labor density, which is expressed by the ratio of net fixed assets to total number of employees. Generally speaking, the degree of environmental pollution caused by capital-intensive industries and non-capital-intensive industries are not the same. (4) Ratio of fixed assets, i.e., the ratio of net fixed assets to total assets. The smaller the ratio is, the stronger the enterprise’s liquidity, and the stronger its innovation initiative. (5) Capital structure, namely asset-liability ratio. The higher the debt ratio is, the greater the debt risk the enterprise assumes, which may limit the cost of environmental protection investment and the effect of technological innovation to a certain extent [41]. (6) Return on assets. This indicator reflects the business environment of the enterprise, and to some extent, it will affect its ability to implement environmental protection measures.

#### 2.3.4. Other Variables

This paper selects two variables as the mediating effect indicators: (1) The quality of environmental management disclosure. The index reflects the corporate environmental management ability, which consists of eight contents, reflecting whether the company is disclosing: the environmental protection concept; the environmental protection objectives; the environmental management system; the environmental protection education and training; the environmental protection special action the environmental protection emergency mechanism; the environmental protection honor or reward; and the “three simultaneity” system. If the answer to one item is “yes”, 1 point will be scored; otherwise, 0 points will be scored. The final score is the quality score of the environmental management disclosure. (2) Number of patent applications: the total number of patent applications filed by a listed company and a subsidiary joint venture company for the year in which the company applied for the patent. The index reflects the ability of companies to fulfill their environmental responsibilities from the technical level.

### 2.4. Data Sources and Descriptive Analysis

To implement the DID model, considering the availability of data, this paper only takes into account the data of 4 years before and after the implementation of the policy, i.e., this paper selects all Chinese A-share listed enterprises that disclose their CSR from 2004 to 2012 as the initial research samples. Due to the loss of corporate performance, its operation and environmental protection practice will change significantly, which makes it fail to fully reflect the decision-making behavior of normal operations in the face of policy implementation. Therefore, this paper follows the standard practice and excludes the companies with ST/ST* from January 2004 to December 2012. In addition, the financial industry differs greatly from entity enterprises in operation and environmental protection operations, which cannot be studied uniformly, therefore, enterprises in the financial industry are excluded from this paper. The annual data in this paper start from 2004, thus excluding the data of listed companies after January 1, 2004. After the above sample processing, the sample size of the empirical research in this paper is 460. The financial and social responsibility data in this paper are from the CSMAR database and the China Research Data Service Platform (CNRDS).

After collecting relevant data, this part also makes descriptive statistical analysis on the variables. Considering that the variables are different among enterprises with different property rights, the samples are also divided into the state-owned-enterprise (SOE) group and the non-state-owned-enterprise (NSOE) group. The differences of CER mean value changes between the treated group and the control group, before and after the policy implementation are reported.

As shown in Table 2, the CER of both the treated group and the control group increased significantly after the policy implementation in 2008, and the CER of the treated group is significantly higher than that of the control group. The change in CER in state-owned enterprises is almost the same as that of the full sample, but the change in CER in non-state-owned enterprises is more significant. The mean value change in the treated group of non-state-owned enterprises is higher than that of the treated group of state-owned enterprises, while the mean value change in the control group of non-state-owned enterprises is lower than that of the control group of state-owned enterprises, and the difference between the treated group and the control group of non-state-owned enterprises is twice that of the state-owned enterprises. The results of descriptive statistics confirm the previous hypothesis.

## 3. Econometric Tests of the Impact of Mandatory CSR Disclosure on CER

### 3.1. Analysis of Regression Results

**Figure 1:**
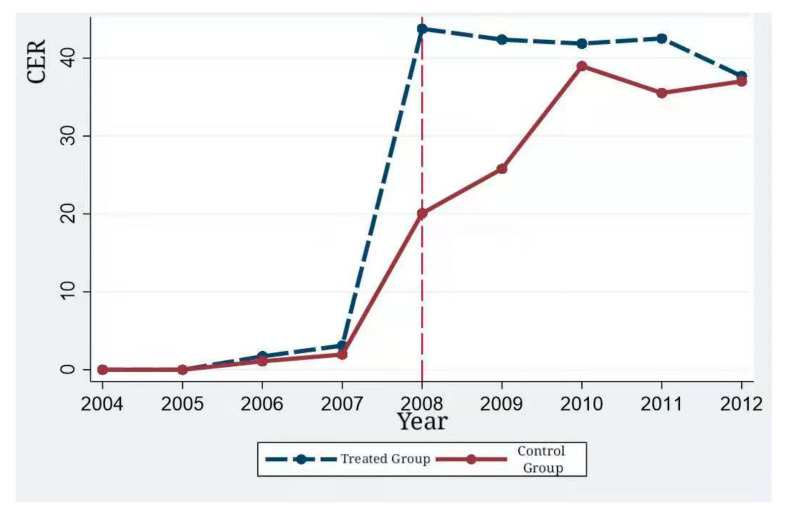
Growth trends of the CER score.

One of the preconditions for the validity of the DID estimation is that the treated group and the control group meet the common trend assumption before being processed [42]. Therefore, in order to verify the appropriateness of the DID model in this paper, we conducted a parallel trend test on the CER scores of the treated group and the control group according to the parallel test procedure. The growth trends of CER scores in the treated group and the control group are shown in Figure 1.

As can be seen from Figure 1, before and after the mandatory disclosure of CSR, the CER scores, as well as the growth trends of the treated group and the control group, are significantly different. Before the release of the mandatory CSR disclosure policy, the CER scores of the control group and the treated group maintained the same growth trend, but after the release of the policy, the CER scores and growth trends of the treated group and the control group changed significantly. Therefore, the result of the DID model is in line with the common trend hypothesis.

According to Model (1), OLS is used to conduct benchmark regression on the impact of a mandatory CSR disclosure policy on CER, and the benchmark DID regression results are shown in Table 3.

In Table 3, column (1) shows the traditional DID regression. Column (2) displays the results of the fixed effect of time and individual. Column (3) displays the results with control variables. The results show that the coefficients of *Treat* *× PL* are 25.33, 9.726 and 9.522, respectively, all of which have significant positive effects under 1%, indicating that the mandatory CSR disclosure policy can significantly improve the CER score of enterprises, and promote enterprises to fulfill their environmental responsibility.

### 3.2. Robustness Test

In order to reach a more reliable conclusion, we conducted two robustness tests. Firstly, a more accurate parallel trend test was conducted for the treated group and the control group. Secondly, we set up a virtual sample by randomly setting the treated group and the control group to conduct the placebo test.

#### 3.2.1. Parallel Trend Test

In the previous part, we made the CER growth trend curve of the treated group and the control group before the benchmark DID regression, and roughly verified that the two groups had the same trend before the policy implementation. In order to verify the parallel trend hypothesis more accurately, the following dynamic effect model is established:

(2) $$CER_{it}=\propto +\sum_{t=2004}^{2012}\beta_{t}Treat_{t\;}\times year^{t}+\sum_{j=1}^{n}\gamma_{j}X_{jit}+\lambda_{i}+v_{t}+\varepsilon_{ikt}$$

where the base year is 2008; *year^t^* is a time dummy variable; *β_t_* represents a series of estimated values from 2004 to 2012; and the definitions of other variables are the same as those of the benchmark regression model (1). Taking the year before the policy, i.e., 2007, as the base period, if the regression coefficients from *β_2004_* to *β_2006_* are not significant, then the treated group and the control group meet the parallel trend test. The specific parameter estimation results are shown in Table 4.

**Figure 2:**
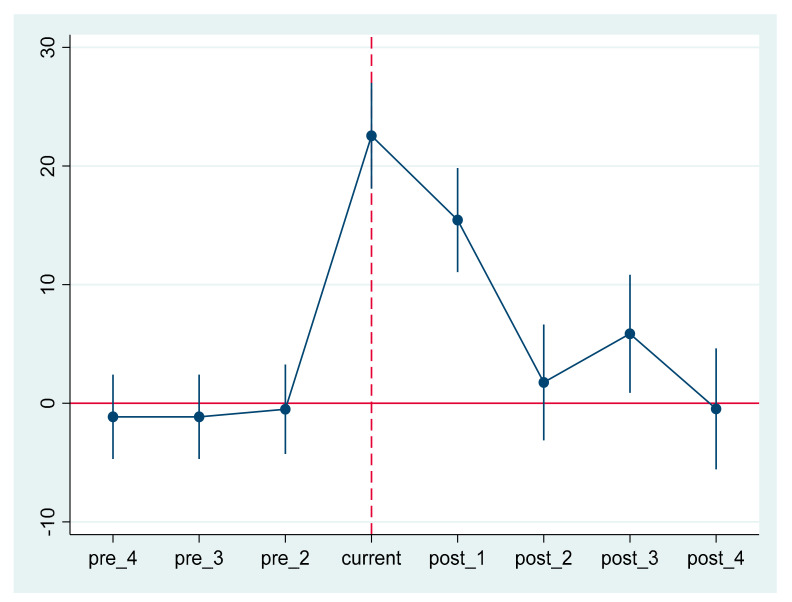
Dynamic effect of the DID model.

The dynamic effects of the estimated values in Table 4 illustrate the parallel trend hypothesis, and the DID model can be used in this quasi-natural experiment. In Table 4, the year before the policy, i.e., 2007, is taken as the base period. Column (1) displays the dynamic effect results of the fixed effects of individual, time and industry, and column (2) displays the results with control variables. *βt* are not significant from 2004 to 2006, indicating that there is no significant difference in environmental responsibility scores between the treated group and the control group before the policy was implemented, satisfying the parallel trend hypothesis. *βt* in 2008 is significantly positive, indicating that the CER score of enterprises that are forced to disclose their CSR in this year is significantly higher than that of enterprises that are not forced to disclose their CSR. The policy can promote enterprises to fulfill their environmental responsibility. Since then, the coefficient has decreased but remained significant, indicating that, compared with 2007, there is still a significant gap between the CER scores of the two groups of enterprises after 2008, as the growth trend shown in Figure 1. The possible reasons are as follows: With the advance of the policy, the number of enterprises subject to mandatory disclosure increased in 2009 and beyond. Meanwhile, voluntary disclosure enterprises in the policy environment strengthen their own environmental responsibility. To better demonstrate the results of the parallel trend test, the point estimates of coefficient *βt* and the corresponding 95% confidence interval estimates are plotted in Figure 2. The figure also reflects the parallel trend of the treated group and the control group, as well as the future trend changes.

#### 3.2.2. Placebo Test

**Figure 3:**
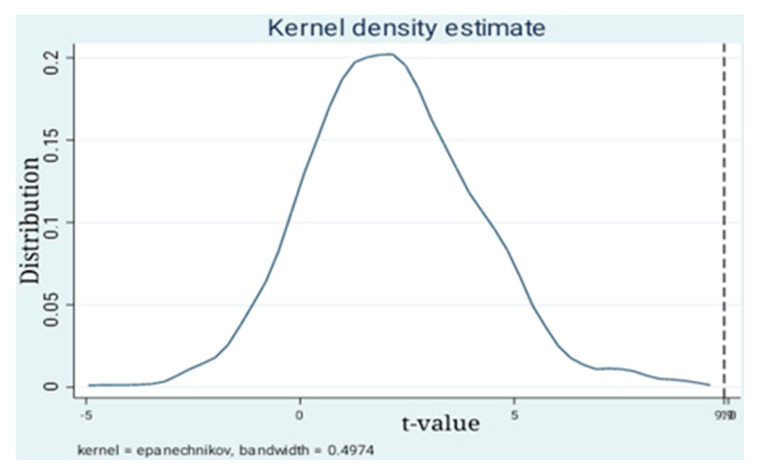
Results of the placebo test. Note: Parameter estimates (dots) and corresponding 95% confidence intervals (lines) are based on Model (2).

In order to further examine the differences in CER scores between enterprises subject to mandatory CSR disclosure and enterprises with non-mandatory CSR disclosure, this study conducts a placebo test by randomly assigning a treated group and a control group. This method ensures that the independent variable *TREL* *×* *PL* has no influence on the CER score. The benchmark model includes 460 sample enterprises, among which 160 are subject to mandatory CSR disclosure (the treated group) and 300 are voluntary disclosure enterprises (the control group). The corresponding numbers of treated groups and control groups are randomly established in this paper, and then regression is performed according to Equation (1). This step was repeated 500 times thereafter. The *t*-value density distribution of these 500 estimates is shown in Figure 3. On the one hand, the t-value presents an inverted U-shaped distribution, which proves that the regression results are robust. On the other hand, the straight dotted line in the figure is the t value of the fundamental regression, which is at the far right of the 500 estimates, also verifying that the fundamental regression is robust.

## 4. The Influence Mechanism of Mandatory CSR Disclosure on CER

Mandatory CSR disclosure has a significant positive effect on CER, but the influence mechanism may have multi-path characteristics. In practice, enterprises tend to strengthen the performance of environmental responsibility from two aspects: management and technology. On the one hand, enterprises should strengthen management and establish scientific working procedures to ensure sufficient execution to achieve strategic goals quickly [43]. On the other hand, enterprises can improve the quality of products and save energy consumption by constantly innovating technology to ensure their core competitiveness and realize sustainable development [44]. Through many experiments and theoretical analysis in the hypotheses, this paper found that mandatory CSR disclosure enhances CER through strengthening green management and Research and Development patent applications. Based on this, this paper uses the mediating effect test to verify H2 by examining the impact of the environmental management disclosure quality and patent application number on the performance of CER.

### 4.1. The Mediating Effect of the Quality of Environmental Management Disclosure

The basic model forms to investigate the mediating effect of environmental management disclosure quality are as follows:

(3) $$CER_{it}=\alpha_{1}+\theta_{1}Treat_{t\;}\times PL_{i}+\sum_{j=1}^{n}\gamma_{j}X_{jit}+\lambda_{i}+v_{t}+\varepsilon_{ikt}$$

(4) $$MNG_{it}=\alpha_{2}+\theta_{2}Treat_{t\;}\times PL_{i}+\sum_{j=1}^{n}\gamma_{j}X_{jit}+\lambda_{i}+v_{t}+\varepsilon_{ikt}$$

(5) $$CER_{it}=\alpha_{3}+\theta_{3}\;\cdot \;Treat_{t\;}\times PL_{i}+\theta_{4}\;\cdot \;MNG_{it}+\sum_{j=1}^{n}\gamma_{j}X_{jit}+\lambda_{i}+v_{t}+\varepsilon_{ikt}$$

where *MNG* represents the quality of an enterprise’s environmental management disclosure. In this part, the stepwise test of regression coefficients is adopted, and the meanings and calculation methods of other variables are consistent with the fundamental DID regression model. *θ1* measures the impact of the mandatory CSR disclosure policy on CER. *θ2* measures the impact of the mandatory CSR disclosure policy on *MNG*. *θ3* measures the direct impact of the mandatory CSR disclosure policy on CER. *θ4* measures the impact of *MNG* on CER. Parameters of models (3)~(5) are estimated, and the results are shown in Table 5.

It can be seen from Table 5 that improving the quality of environmental management disclosure plays a mediating role in the influence mechanism of mandatory CSR disclosure on CER. Specifically, column (1) in Table 5 verifies the direct impact of mandatory CSR disclosure on CER; column (2) verifies the impact of mandatory CSR disclosure on the quality of corporate environmental management disclosure. The regression coefficient is 0.281 and significantly positive, indicating that mandatory CSR disclosure does lead to an increase in the quality of environmental management disclosure. Column (3) shows that the impact coefficients of mandatory CSR disclosure on CER and environmental management disclosure quality are significantly positive, indicating that the environmental management disclosure quality plays a partial mediating role in the effect of mandatory CSR disclosure on CER. Meanwhile, the Sobel test is used to verify that the mediating effect is robust [45,46].

### 4.2. Mediating Effect of the Number of Patents Application

The basic model forms to investigate the mediating effect of the number of patent applications are as follows:

(6) $$CER_{it}=\alpha_{4}+\beta_{1}Treat_{t\;}\times PL_{i}+\sum_{j=1}^{n}\gamma_{j}X_{jit}+\lambda_{i}+v_{t}+\varepsilon_{ikt}$$

(7) $$Patent_{it}=\alpha_{5}+\beta_{2}Treat_{t\;}\times PL_{i}+\sum_{j=1}^{n}\gamma_{j}X_{jit}+\lambda_{i}+v_{t}+\varepsilon_{ikt}$$

(8) $$CER_{it}=\alpha_{6}+\beta_{3}\;\cdot \;Treat_{t\;}\times PL_{i}+\beta_{4}\;\cdot \;Patent_{it}+\sum_{j=1}^{n}\gamma_{j}X_{jit}+\lambda_{i}+v_{t}+\varepsilon_{ikt}$$

where *Patent* represents the number of an enterprise’s patent applications. The meanings and calculation methods of other variables are consistent with the fundamental DID regression model. *β1* measures the impact of a mandatory CSR disclosure policy on CER. *β2* measures the impact of a mandatory CSR disclosure policy on *Patent*. *β3* measures the direct impact of a mandatory CSR disclosure policy on CER. *β4* measures the impact of the number of an enterprise’s patent applications on CER. Parameters of models (6)–(8) are estimated, and the results are shown in Table 6.

It can be seen from Table 6 that the number of corporate patent applications plays a mediating role in the influence mechanism of mandatory CSR disclosure on CER. Specifically, column (1) in Table 6 verifies the direct impact of mandatory CSR disclosure on CER, while column (2) verifies the impact of mandatory CSR disclosure on the number of corporate patents application. The regression coefficient is 79.98 and significantly positive, which indicates that mandatory CSR disclosure does lead to an increase in the number of corporate patents application. Column (3) shows that the impact coefficients of mandatory CSR disclosure on CER and the number of corporate patents application are significantly positive, indicating that the number of corporate patent applications plays a partial mediating role in the effect of mandatory CSR disclosure on CER. Meanwhile, the Sobel test is used to verify that the mediating effect is robust.

Combining the above two parts, we can draw a conclusion that the empirical results indicate that the impact of mandatory CSR disclosure on CER is to some extent realized by improving the quality of enterprise environmental management and increasing the number of patent applications.

## 5. Heterogeneity Analysis

### 5.1. Heterogeneity Caused by Different CER Levels

Different CER levels reflect different levels of legal awareness, social evaluation, low-carbon technology and output, and green management [47]. Due to the differences in enterprises’ characteristics, the dispersion degree of CER is great, and the estimated results may be affected by extreme values. This part refers to the practice of Li et al. [32], adopts the benchmark regression Equation (1), as well as quantile regression to study the impact of a mandatory CSR disclosure policy on CER scores at different levels to verify Hypothesis 3. The quantile regression model is used to conduct the parameter estimation, and the results are shown in Table 7.

It can be concluded from Table 7 that there is an inverted U-shaped relationship between the CER level and mandatory CSR disclosure effect. Table 7 shows the regression results of CER scores at the 10% to 90% quantile levels. It can be seen that the mandatory disclosure policy has a significant impact on CER scores from 10% to 90%. The coefficient first increases, reaches the maximum at 30%, and then decreases, which is consistent with Hypothesis 3. On the whole, mandatory disclosure policies can promote the CER, but when the level of CER is very low, the environmental problems of enterprises tend to be more serious, with much room for improvement, so the mandatory disclosure of CSR forces enterprises to carry out corresponding innovation in order to meet the disclosure requirements, and improve their level of environmental responsibility at a faster speed. When an enterprise’s CER increases to the level of 0.3 or above, its development will encounter a bottleneck, and the growth rate of its CER will slow down due to management and technical limitations.

### 5.2. Heterogeneity Caused by Different Corporate Scales

Past studies have shown that companies of different sizes usually perform differently [48,49,50]. Fortunati et al., analyzed that small and medium sized enterprises in the Italian agri-food industry could provide an adequate level of circular strategies and social responsibility practices [51]. This part adopts the benchmark regression Equation (1), as well as the quantile regression of corporate scale, to study the impact of a mandatory CSR disclosure policy on CER scores to verify Hypothesis 3. The quantile regression model is used to conduct parameter estimation, and the results are shown in Table 8.

It can be seen from Table 8 that the promotion effect of a compulsory CSR disclosure policy on CER levels is heterogeneous at different corporate scales. In short, the promotion effect of a compulsory CSR disclosure policy on CER increases with the increase in the corporate scale. In the sample group of about 20% of the largest enterprises, the promotion effect of the policy is not significant. This is due to the number of large-scale enterprises in the treated group and in the control group being unbalanced, which leads to the bias of results. When the policy is promulgated, large-scale enterprises have to make stronger responses to meet their regulatory requirements and social reputation, due to their social influence.

### 5.3. Heterogeneity Caused by XSD Different Property Rights Structure

Different property rights reflect different stakeholders and different ways of making decisions [52]. Therefore, in view of the difference in property rights structures, this paper continues to discuss the impact of mandatory disclosure of CSR on CER. In 2008, when the policy was issued, there were 342 state-owned sample enterprises (SOEs) and about 118 non-state-owned sample enterprises (NSOEs), which were respectively tested according to the benchmark regression Equation (1), and the results are shown in Table 9.

It can be concluded from Table 9 that the impact of mandatory CSR disclosure on CER is heterogeneous in terms of different property rights structures. Table 9 shows the impact of mandatory disclosure policies on the CER scores of SOEs and NSOEs. Columns (1) and (3) show the regression results of SOEs and NSOEs, respectively. Columns (2) and (4) show results with control variables based on the above results, respectively. We can see that the policy has a significant positive impact on the CER performance of enterprises with different property rights structures. However, the regression coefficient of non-state-owned enterprises is about twice that of state-owned enterprises, indicating that the promotion effect of the policy on non-state-owned enterprises is greater than that on state-owned enterprises. One of the stakeholders of state-owned enterprises is the state (investor), while the investor of non-state-owned enterprises is the private sector, which is more focused on economic interests, and, due to the mandatory CSR disclosure policies, non-SOEs are forced to take environmental objectives into account, so the CER changes are more dramatic than those in SOEs that reflect the will of the state to some extent.

## 6. Conclusions

Faced with environmental deterioration, China pays special attention to the popularization of environmental awareness and the implementation of environmental measures, especially in enterprises. On the basis of voluntary CSR disclosure, mandatory disclosure policies for three types of enterprises are implemented, namely, the sample companies of “Shanghai Stock Exchange Corporate Governance Sector”, the companies issuing overseas listed foreign shares, and financial companies, in order to reduce the opportunities for enterprises to hide environmental pollution and reduce the cost of government supervision. In this context, this paper uses the DID model to test the impact of mandatory CSR disclosure policies on corporate environmental responsibility (CER). The main conclusions are as follows:

First, mandatory CSR disclosure policies can promote the fulfillment of CER by enterprises. The CER scores of enterprises subject to mandatory disclosure are higher than those of voluntary disclosure enterprises. However, this policy has a short-term effect. In the long run, the CER gap between mandatory disclosure enterprises and voluntary disclosure enterprises gradually narrows. With the promotion of the policy, the number of enterprises subject to mandatory disclosure in 2009 and later increases; at the same time, the voluntary disclosure enterprises strengthen their own environmental responsibility in the policy environment, and the threshold of the whole society has also been raised.

Second, after the implementation of mandatory CSR disclosure policies, enterprises can improve their CER level through two channels: improving the quality of enterprise environmental management and increasing the number of patent applications. In order to meet the disclosure requirements, enterprises will strengthen the implementation of environmental responsibility from two aspects, namely management and technology. From the management level, enterprises will increase the emphasis on their own environmental protection concept and environmental protection goals, and establish an environmental management system, environmental knowledge education and training, and an environmental emergency response mechanism to regulate their own behavior. From the technical level, through the investment of green technology innovation, enterprises can produce more green patents and realize their sustainable development strategy.

Third, the heterogeneity of the effect of a mandatory CSR disclosure policy on CER is reflected in three aspects: CER levels, corporate scales, and property rights structure. In terms of the CER level, there is an inverted U-shaped relationship between the CER level and the mandatory CSR disclosure effect. In terms of the corporate scale, mandatory disclosure of CSR plays a greater role in large-scale enterprises. In terms of property rights, mandatory disclosure of CSR plays a greater role in non-state-owned enterprises. Specifically, a mandatory CSR disclosure policy has different impacts on enterprises with different levels of CER. When CER is at or below the 30% quantile level, the promoting effect of the mandatory CSR disclosure policy increases; when CER is above the 30% quantile level, the promoting effect decreases, presenting an inverted U-shaped change. A mandatory CSR disclosure policy promotes change in non-state-owned enterprises better than in state-owned enterprises. Shareholders of state-owned enterprises will pay more attention to sustainable strategic development, while non-state-owned enterprises will pay more attention to economic benefit maximization, so their shareholders pay less attention to environmental issues than shareholders of state-owned enterprises. The implementation of the mandatory CSR disclosure policy makes shareholders of non-state-owned enterprises pay more attention to the performance of environmental responsibility. Therefore, the CER improvement level of non-state-owned enterprises is higher than that of state-owned enterprises.

The conclusions provide following implications: First, policy makers can use mandatory policies in the field of corporate environmental responsibility to achieve green production and green management. Second, enterprises should concentrate on the quality of enterprise environmental management and number of patent applications, which are key to improving their CER levels. In the absence of mandatory policies, enterprises can also improve the above two parts through independent efforts to gradually realize environmental responsibility. Finally, governments should formulate appropriate policy strategies in the face of different types of corporate entities and pay more attention to those with extreme CER levels, as well as small and medium size and state-owned enterprises.

Furthermore, as corporate social responsibility is an important and complex issue, it is not enough to consider only the environmental responsibility. Future research should focus on multi-dimensional research of corporate social responsibility [53]. The existing research on mandatory disclosure policies also lacks multi-dimensional consideration, which will be the main direction of our future research.

## Figures and Tables

**Table 1:** Corporate environmental responsibility scoring system.

Dimensions	Indicators
Legal awareness (10%)	1. Whether following the GRI “Sustainability Reporting Guidelines” (1, 0); 2. Whether disclosing environment and sustainable development (1, 0); 3. Whether Environmental penalties are imposed (0, 1)
Social assessment (15%)	1. Whether receiving environmental recognition (1, 0); 2. Whether having environmental advantages (1, 0)
Environment- friendly outputs (25%)	1. Whether having a circular economy (1, 0); 2. Whether having environmentally beneficial products (1, 0); 3. Whether having pollution emissions (0, 1)
Low-carbon technologies (25%)	1. Whether conserving energy (1, 0); 2. Whether having measures to reduce processing wastes (1, 0)
Green management (25%)	1. Whether there is a third-party inspection (1, 0); 2. Whether adopting green office (1, 0)

**Table 2:** Descriptive statistics of the variables.

Variable	Observations	Mean Value	Standard Deviation	Minimum Value	Maximum Value
CER	4140	18.94975	23.11495	0	92.50000
Corporate scale	4140	22.25129	1.30309	16.18471	27.85198
Corporate age	4140	12.52278	4.43182	2.00000	31.00000
Fixed asset ratio	4140	0.29179	0.19889	0	0.93634
Capital structure	4140	0.52593	0.22456	0.02910	3.88615
Return on assets	4140	0.04371	0.11799	−4.16097	2.14273
Capital labor density	4140	2069.40000	1202.84100	1.00000	4153.00000
Number of patents application	4140	50.41715	278.21780	0	6437
Quality of environmental management disclosure	4140	0.17343	0.80909	0	8
Changes in CER mean before and after policy			Full sample		
			Treated group	Control group	Difference
			+40.44510	+32.63288	7.81222
Changes in CER mean before and after policy			State-owned enterprises		
			Treated group	Control group	Difference
			+39.56243	+31.91209	7.65033
Changes in CER mean before and after policy			Non-State-owned enterprises		
			Treated group	Control group	Difference
			+44.55052	+28.14369	16.40683

**Table 3:** DID regression results.

Items	(1)	(2)	(3)
	CER	CER	CER
*Treat × PL*	25.33 *** (25.25)	9.726 *** (9)	9.522 *** (8.67)
_cons	16.32 *** (45.24)	10.02 ** (2.26)	−59.23 *** (−3.99)
Control variables	no	no	yes
Time fixed effects	no	yes	yes
Individual fixed effects	no	yes	yes
N	4140	4140	4140
R-sq	0.110	0.667	0.670

** *p* < 0.05, *** *p* < 0.01, *t* values are in brackets.

**Table 4:** Dynamic effects of mandatory CSR disclosure policy on CER.

Items	(1)	(2)
	CER	CER
Treat × year^2004^	−1.140	−1.002
	(−0.630)	(−0.560)
Treat × year^2005^	−1.140	−0.919
	(−0.63)	(−0.52)
Treat × year^2006^	−0.502	−0.564
	(−0.26)	(−0.30)
Treat × year^2007^	omitted	omitted
Treat × year^2008^	22.550 ***	22.420 ***
	(9.90)	(9.88)
Treat × year^2009^	15.450 ***	15.340 ***
	(6.900)	(6.850)
Treat × year^2010^	1.760	1.587
	(0.71)	(0.64)
Treat × year^2011^	5.860 *	5.662 *
	(2.31)	(2.27)
Treat × year^2012^	−0.468	−0.579
	(−0.18)	(−0.23)
Constant term	10.800 *	−48.390 ***
	(2.13)	(−4.20)
Control variables	no	yes
Time fixed effects	yes	yes
Individual fixed effects	yes	yes
N	4140	4140
R-sq	0.679	0.682

* *p* < 0.10, *** *p* < 0.01, *t* values are in brackets.

**Table 5:** Mediating effect results of environmental management disclosure quality.

Items	(1)	(2)	(3)
	CER	MNG	CER
*Treat × PL*	9.357 ***	0.281 ***	9.046 ***
	(8.52)	(4.36)	(8.25)
MNG			1.110 ***
			(2.97)
Constant term	−62.360 ***	−0.282	−62.050 ***
	(−4.19)	(−0.37)	(−4.18)
Control variables	yes	yes	yes
Time fixed effects	yes	yes	yes
Individual fixed effects	yes	yes	yes
N	4140	4140	4140
R-sq	0.672	0.277	0.673

*** *p* < 0.01, *t* values are in brackets.

**Table 6:** Results of mediating effect of the number of patents application.

Items	(1)	(2)	(3)
	CER	Patent	CER
*Treat × PL*	9.357 ***	79.980 ***	8.997 ***
	(8.52)	(3.76)	(8.15)
Patent			0.005 ***
			(3.21)
Constant term	−62.360 ***	−843.900 ***	−56.020 ***
	(−4.19)	(−5.98)	(−4.76)
Control variables	Yes	yes	yes
Time fixed effects	Yes	yes	yes
Individual fixed effects	Yes	yes	yes
N	4140	4140	4140
R-sq	0.672	0.707	0.673

*** *p* < 0.01, *t* values are in brackets.

**Table 7:** CER estimation results at different quantile levels.

Items	(1)	(2)	(3)	(4)	(5)	(6)	(7)	(8)	(9)
	10th CER	20th CER	30th CER	40th CER	50th CER	60th CER	70th CER	80th CER	90th CER
*Treat × PL*	15.00 ***	27.50 ***	35.00 ***	33.52 ***	23.27 ***	17.9 ***	13.44 ***	9.56 ***	10.99 ***
	(0.416)	(0.129)	(0.133)	(1.544)	(2.042)	(1.672)	(1.842)	(1.888)	(1.977)
Constant term	0	0	0	−23.63 **	−90.50 ***	−126.80 ***	−144.70 ***	−142.50 ***	−111.50 ***
	(2.124)	(0.659)	(0.676)	(7.881)	(10.420)	(8.532)	(9.402)	(9.639)	(10.090)
Control variables	yes	yes	yes	yes	yes	yes	yes	yes	yes
Time fixed effects	yes	yes	yes	yes	yes	yes	yes	yes	yes
Individual fixed effects	yes	yes	yes	yes	yes	yes	yes	yes	yes
N	4140	4140	4140	4140	4140	4140	4140	4140	4140
Pseudo R2	0.0149	0.0658	0.0982	0.1242	0.2028	0.2608	0.2337	0.1885	0.1489

** *p* < 0.05, *** *p* < 0.01, *t* values are in brackets.

**Table 8:** CER estimation results of different corporate scales.

Items	(1)	(2)	(3)	(4)	(5)
	20th Scale	40th Scale	60th Scale	80th Scale	100th Scale
*Treat × PL*	4.578	3.403	7.706 ***	11.48 ***	1.69
	(0.81)	(0.77)	(3.55)	(5.71)	(0.83)
Constant term	2.318	−10.29 *	−2.53	7.578	8.182 **
	(0.56)	(−1.77)	(−0.57)	(1.27)	(2.06)
Control variables	yes	yes	yes	yes	yes
Time fixed effects	yes	yes	yes	yes	yes
Individual fixed effects	yes	yes	yes	yes	yes
N	828	828	828	828	828
R-sq	0.543	0.65	0.668	0.703	0.767

* *p* < 0.10, ** *p* < 0.05, *** *p* < 0.01, *t* values are in brackets.

**Table 9:** Estimation results of different property rights structures.

Items	(1)	(2)	(3)	(4)
	CER (SOEs)	CER (SOEs)	CER (NSOEs)	CER (NSOEs)
*Treat* *× PL*	7.499 ***	6.911 ***	16.300 ***	15.290 ***
	(6.03)	(5.61)	(6.15)	(5.87)
Constant term	10.410 *	−80.470 ***	4.050	−71.060 ***
	(2.25)	(−5.34)	(0.80)	(−3.90)
Control variables	no	yes	no	yes
Time fixed effects	yes	yes	yes	yes
Individual fixed effects	yes	yes	yes	yes
N	3080	3080	1060	1060
R-sq	0.674	0.681	0.672	0.677

* *p* < 0.10, *** *p* < 0.01, *t* values are in brackets.

## Data Availability

Not applicable.
